# mRNA markers associated with malignant pleural effusion

**DOI:** 10.1038/s41598-023-32872-2

**Published:** 2023-04-24

**Authors:** Shih-Chang Hsu, Shan-Yueh Chang, Yi-Ting Hwang, Harn-Jing Terng, Chen-Liang Tsai, Chih-Hao Shen, Shau Ku Huang, Chih‑Feng Chian

**Affiliations:** 1 Department of Emergency, School of Medicine, College of Medicine, Taipei Medical University, Taipei, Taiwan, ROC; 2 Emergency Department, Wan Fang Hospital, Taipei Medical University, Taipei, Taiwan, ROC; 3 Division of Pulmonary and Critical Medicine, Department of Internal Medicine, Tri-Service General Hospital, National Defense Medical Center, Taipei, Taiwan, ROC; 4 Department of Statistics, National Taipei University, Taipei, Taiwan, ROC; 5 Advpharma, Inc, Taipei, Taiwan, ROC; 6 National Institute of Environmental Health Sciences, National Health Research Institutes, Zhunan, Taiwan, ROC; 7 Johns Hopkins Asthma and Allergy Center, Johns Hopkins University School of Medicine, Baltimore, USA

**Keywords:** Biomarkers, Diseases, Medical research

## Abstract

Malignant pleural effusions (MPE) commonly result from malignant tumors and represent advanced-stage cancers. Thus, in clinical practice, early recognition of MPE is valuable. However, the current diagnosis of MPE is based on pleural fluid cytology or histologic analysis of pleural biopsies with a low diagnostic rate. This research aimed to assess the diagnostic ability of eight previously identified Non-Small Cell Lung Cancer (NSCLC)-associated genes for MPE. In the study, eighty-two individuals with pleural effusion were recruited. There were thirty-three patients with MPE and forty-nine patients with benign transudate. mRNA was isolated from the pleural effusion and amplified by Quantitative real-time PCR. The logistic models were further applied to evaluate the diagnostic performance of those genes. Four significant MPE-associated genes were discovered in our study, including Dual-specificity phosphatase 6 (DUSP6), MDM2 proto-oncogene (MDM2), Ring finger protein 4 (RNF4), and WEE1 G2 Checkpoint Kinase (WEE1). Pleural effusion with higher expression levels of MDM2 and WEE1 and lower expression levels of RNF4 and DUSP6 had a higher possibility of being MPE. The four-gene model had an excellent performance distinguishing MPE and benign pleural effusion, especially for pathologically negative effusions. Therefore, the gene combination is a suitable candidate for MPE screening in patients with pleural effusion. We also identified three survival-associated genes, WEE1, Neurofibromin 1 (NF1), and DNA polymerase delta interacting protein 2 (POLDIP2), which could predict the overall survival of patients with MPE.

## Introduction

Pleural effusions (PEs) are common clinical complications caused by various diseases, such as infection, heart failure, pericardial diseases, cirrhosis, and malignant tumors^1^. Malignant pleural effusions (MPEs) are primarily due to metastatic cancer and indicate an advanced stage of the disease with a poor prognosis^2^. The median survival time of patients with PE originating from breast or lung cancer ranges from two to six months^3^. However, with recent advances in precision medicine and relevant treatment, the median survival time of lung cancer with MPE is increased to around 16.4 months reported in a German study published in 2023^4^. Concerning patients with non-small cell lung cancer (NSCLC), the patient with MPE is staged as IV with a 1-year survival rate of 12.6%^5^. In addition, the 5-year survival rate of stage IV NSCLC is about 5.8% in the US^5,6^.

The gold standard of MPE diagnosis is made by thoracentesis with analysis of pleural fluid cytology or histologic analysis of pleural biopsies. The diagnostic rate for positive malignancy by pleural fluid cytology obtained on initial thoracentesis is 40–60%^7^. Therefore, a pleural biopsy is recommended when pleural fluid cytology is negative and clinically with high suspicion of malignancy^8^. However, a pleural biopsy is an invasive procedure associated with morbidity and mortality and is technically demanding and high cost. Several conventional cancer biomarkers have been utilized to differentiate MPE and benign pleural effusion, but still with low diagnostic utility, including carcinoembryonic antigen (CEA), carbohydrate antigens 125(CA125), carbohydrate antigen 19-9 (CA19-9), carbohydrate antigen 15-3 (CA15-3), cytokeratin 19 (CK19), and neuron-specific enolase (NSE)^9^. Therefore, identifying a reliable, less invasive, and economical tool for diagnosing MPE has excellent clinical value.

Recently, a few molecular markers have demonstrated better sensitivity in diagnosing NSCLC and MPE than immunocytochemistry^10–13^. The mRNA of conventional biomarker genes such as Lung-specific X (LUNX) and vascular endothelial growth factor (VEGF) were evaluated to distinguish between MPE and benign pleural effusion^14–16^. Also, microRNAs (miRNAs) such as miR-182-5p and miR-34a-5p were suggested to be used for the diagnosis^17^. However, the diagnostic performance of current mRNA markers for MPE detection is suboptimal, especially for those effusions that are cytology negative. In clinical practice, breast and lung cancer account for more than 50% of primary tumors metastatic to the pleural space^18^, and approximately 85% of lung cancer cases are NSCLC^19^. Since MPE diagnosis alters cancer staging and greatly influences treatment options, the demand for sensitive detection of MPE is largely unmet.

This study investigated the expression levels of eight NSCLC-associated genes, including Dual-specificity phosphatase 6 (DUSP6), Eukaryotic translation initiation factor 2 subunit gamma (EIF2S3), Growth factor receptor bound protein 2 (GRB2), MDM2 proto-oncogene (MDM2), Neurofibromin 1 (NF1), DNA polymerase delta interacting protein 2 (POLDIP2), Ring finger protein 4 (RNF4), and WEE1 G2 Checkpoint Kinase (WEE1)^20^, for their ability to differentiate between MPE and benign pleural effusion. Initially, we evaluated the diagnosis power of each gene. Multiple markers could improve test sensitivity and are commonly used in cancer diagnostic and prognostic tool development^15,21,22^. Hence, we combined the genes which have the acceptable ability in MPE diagnosis for further multivariate analyses. We utilized logistic models to evaluate the diagnostic performance of those genes and selected the best gene combination for distinguishing between MPE and benign pleural effusion. Finally, Cox regression was used to analyze the association between each expressing gene and survival time.

## Results

### Data description for PE samples

All pleural effusions were newly diagnosed. In this study, four of thirty-three patients received chemotherapy before enrollment. The other twenty-nine patients were treatment-naïve at the time of diagnosis of MPE. However, the analysis of gene expressions in the pleural fluid was done at the initial diagnosis of MPE in all patients. We collected eighty-two effusion samples, while thirty-three (40.24%) samples were classified as malignant pleural effusion (MPE) from patients with cancer in stage IV, and forty-nine (59.76%) were transudates (as control) from patients with congestive heart disease, cirrhosis of the liver, and nephrotic syndromes. The proportion of males in the MPE group (48.48%) was slightly lower than that in the control group (57.14%). A detailed description of the study samples is listed in Table 1.

**Table 1:** Demographics and characteristics of the study group.

Characteristic	Total	Control	MPE	*P*-value
Male, n (%)^a^	44 (53.66)	28(57.14)	16 (48.48)	0.441
Age, year^b^	74.6 ± 14.6	78.2 ± 12.5	69.4 ± 15.8	0.006*
*Diseases*				
Lung cancer				
Adenocarcinoma	–	–	26	–
Squamous cell carcinoma	–	–	2	–
Breast cancer	–	–	3	–
Thymic carcinoma	–	–	1	–
Gingival cancer	–	–	1	–
Congestive heart failure	–	40	–	–
Cirrhosis of liver	–	8	–	–
Nephrotic syndrome	–	1	–	–

^a^The chi-square test was used.

^b^The two-independent sample T was used.

*P-value < 0.05.

### Bivariate Analysis of MPE-associated factors

A case–control study was conducted on the MPE group (33 cases) and the transudate group (49 controls). The relative expression of each investigated gene was applied to analyze the bivariate association and logistic regression. Each gene expression data was obtained by normalization with the expression of the reference gene, HPRT1, according to the data process for relative quantitative real-time PCR assay. The overall mean expression for DUSP6, EIF2S3, NF1, and POLDIP2 was higher than for the reference gene, whereas the other four genes were lower. Evaluation of bivariate associations between the mean expression of each gene in the MPE group and control group was provided in Table 2. The expressions of the four genes, DUSP6, GRB2, RNF4, and WEE1, were statistically significant differences between the MPE case and the control group. Only the mean expression of the WEE1 gene for MPE cases was higher than that for the control.

**Table 2:** Bivariate analyses for the relative gene expression of the molecular markers in malignant pleural effusions.

	Total		Control		MPE		*P*-value^§^
Variable	Mean	STD^$^	Mean	STD	Mean	STD	
DUSP6	0.30	1.04	0.58	0.77	− 0.12	1.24	0.006*
EIF2S3	2.02	0.65	2.02	0.39	2.01	0.91	0.943
GRB2	− 0.08	1.07	0.40	0.71	− 0.80	1.11	< .001*
MDM2	− 0.62	0.79	− 0.60	0.67	− 0.66	0.95	0.777
NF1	0.85	0.53	0.84	0.32	0.88	0.75	0.735
POLDIP2	0.58	0.46	0.60	0.37	0.54	0.57	0.559
RNF4	− 0.30	0.48	− 0.19	0.28	− 0.46	0.64	0.029*
WEE1	− 1.21	1.15	− 1.63	0.88	− 0.59	1.24	< .001*

^§^ The two-independent sample T was used, *P-value < 0.05, *STD* standard deviation.

### MPE-associated gene signature

Four genes, DUSP6, MDM2, RNF4, and WEE1, were selected as highly significant MPE-associated factors using the backward selection for the logistic regression controlling for age and sex (Table 3). PE samples with higher expression levels of MDM2 and WEE1 and lower expression levels of RNF4 and DUSP6 were more likely to be MPE cases. For each unit increase in the expression of WEE1, the odds of being an MPE case were 6.13 times higher. For each unit increase in the expression of MDM2, the odds of being an MPE case were 3.25 times higher. For every unit in the expression of RNF4 and DUSP6, the odds ratios of being MPE case in the expression of RNF4 and DUSP6 declined by 99% and 74%, respectively. Based on the standardized estimate and *p-*value, the most significant predictor was RNF4, followed by WEE1, DUSP6, and MDM2.

**Table 3:** Gene signature for classification model based on the logistic regression.

	OR	Lower CI	Upper CI	SE	StdEst	*P*-value
Age	0.97	0.93	1.02	0.02	− 0.21	0.288
Male vs Female	0.83	0.23	3.08	0.67	− 0.05	0.785
MDM2	3.25	1.01	10.38	0.59	0.51	0.047*
WEE1	6.13	2.07	18.21	0.56	1.15	0.001*
RNF4	0.01	0.00	0.15	1.63	− 1.33	0.002*
DUSP6	0.26	0.09	0.73	0.53	− 0.77	0.011*

*P-value < 0.05.

*OR* odds ratio, *CI* confidence interval, *SE* standard error, *StdEs* Standardized estimates of coefficient.

**Figure 1:**
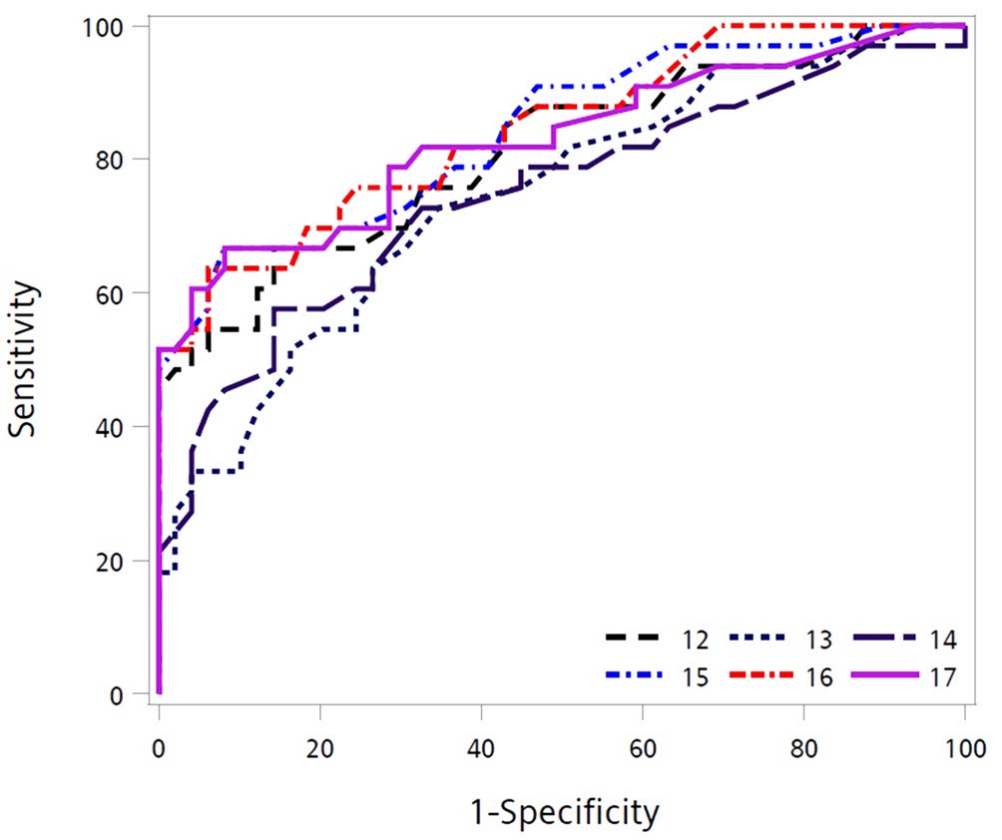
ROCs for three- and four-gene models; Model 12: MDM2/RNF4/WEE1; Model 13: DUSP6/MDM2/WEE1; Model 14: DUSP6/MDM2/RNF4; Model 15: DUSP6/RNF4/WEE1; Model 16: DUSP6/MDM2/RNF4/WEE1; Model 17: DUSP6/MDM2/RNF4/WEE1/Age/Sex.

To understand the combined diagnosis effects, we further conducted logistic models with one, two, and three significant genes to compare with the four-gene model (Table 4). The AUC for the WEE1-gene model (0.76) was the highest among single-gene models. The model containing WEE1/RNF4-combination resulted in higher AUC for two-gene (0.84) and three-gene (0.85 and 0.88) models. The four-gene model with and without controlling age and sex achieved the same AUC (0.89). The ROC for three- and four-gene models were presented in Fig. 1.

**Table 4:** AUCs of logistic models.

Model ID	Gene combination	AUC
Single-gene models		
M2	MDM2	0.52
M3	WEE1	0.76
M4	RNF4	0.65
M5	DUSP6	0.69
Two-gene models		
M6	MDM2/WEE1	0.75
M7	MDM2/RNF4	0.66
M8	DUSP6/MDM2	0.70
M9	RNF4/WEE1	0.84
M10	DUSP6/WEE1	0.79
M11	DUSP6/RNF4	0.73
Three-gene models		
M12	MDM2/RNF4/WEE1	0.85
M13	DUSP6/MDM2/WEE1	0.79
M14	DUSP6/MDM2/RNF4	0.79
M15	DUSP6/RNF4/WEE1	0.88
Four-gene models		
M16	DUSP6/MDM2/RNF4/WEE1	0.89
M17	DUSP6/MDM2/RNF4/WEE1/Age/Sex	0.89

**Figure 2:**
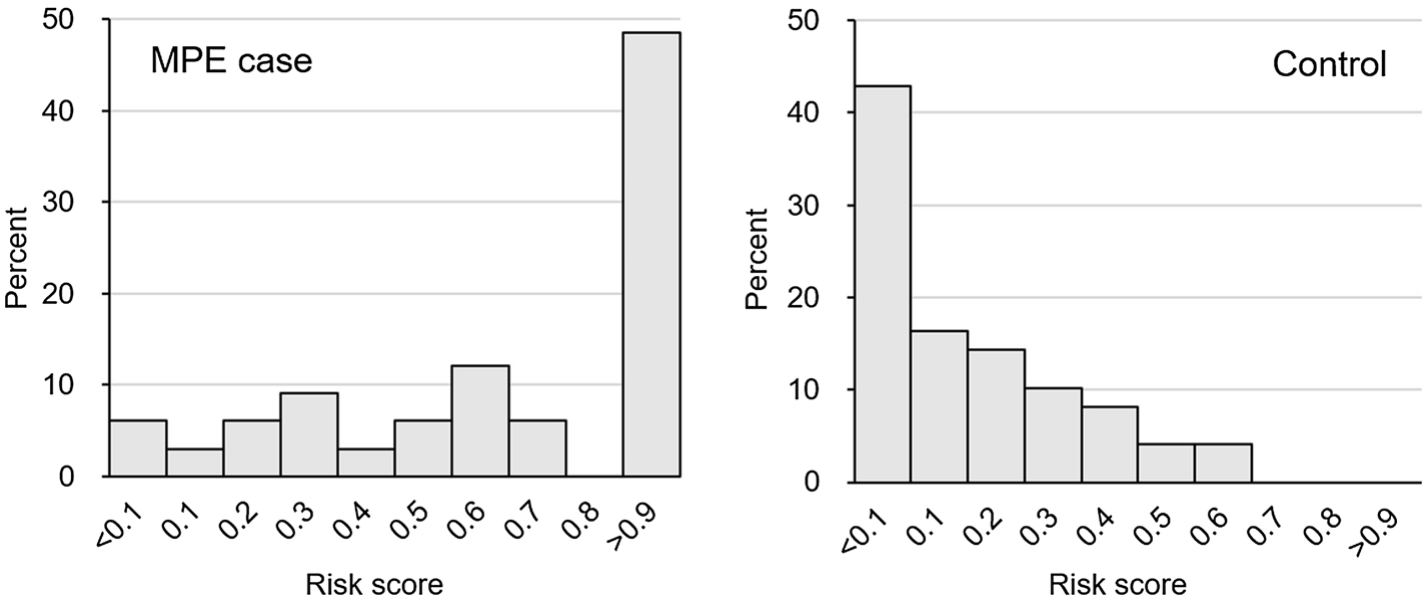
Histogram of risk score of samples (in percent).

The performance for the four-gene model yielded a sensitivity of 75.8%, a specificity of 89.8%, and an accuracy of 84.15% for classification between the MPE case and control. In addition, the distribution of risk scores for PE samples could be graphically presented as a histogram (Fig. 2). The majority (48.5%) of MPE cases had a risk score greater than 0.9, while 42.9% of control had a risk score less than 0.1.

### Subgroups of MPE cases and detection sensitivities

MPE cases were further stratified into subgroups according to cancer type and cytological examination results to evaluate the sensitivity of the four-gene model for the subgroup of MPE cases. The sensitivity was 75% for lung cancer and 80% for other cancer. Moreover, four of five (80%) cytology negative MPE were detected, while the sensitivity was 75% for positive cytology MPE. The four-gene model identified all three (100%) MPE without any pathological evidence.

### Survival analysis

**Figure 3:**
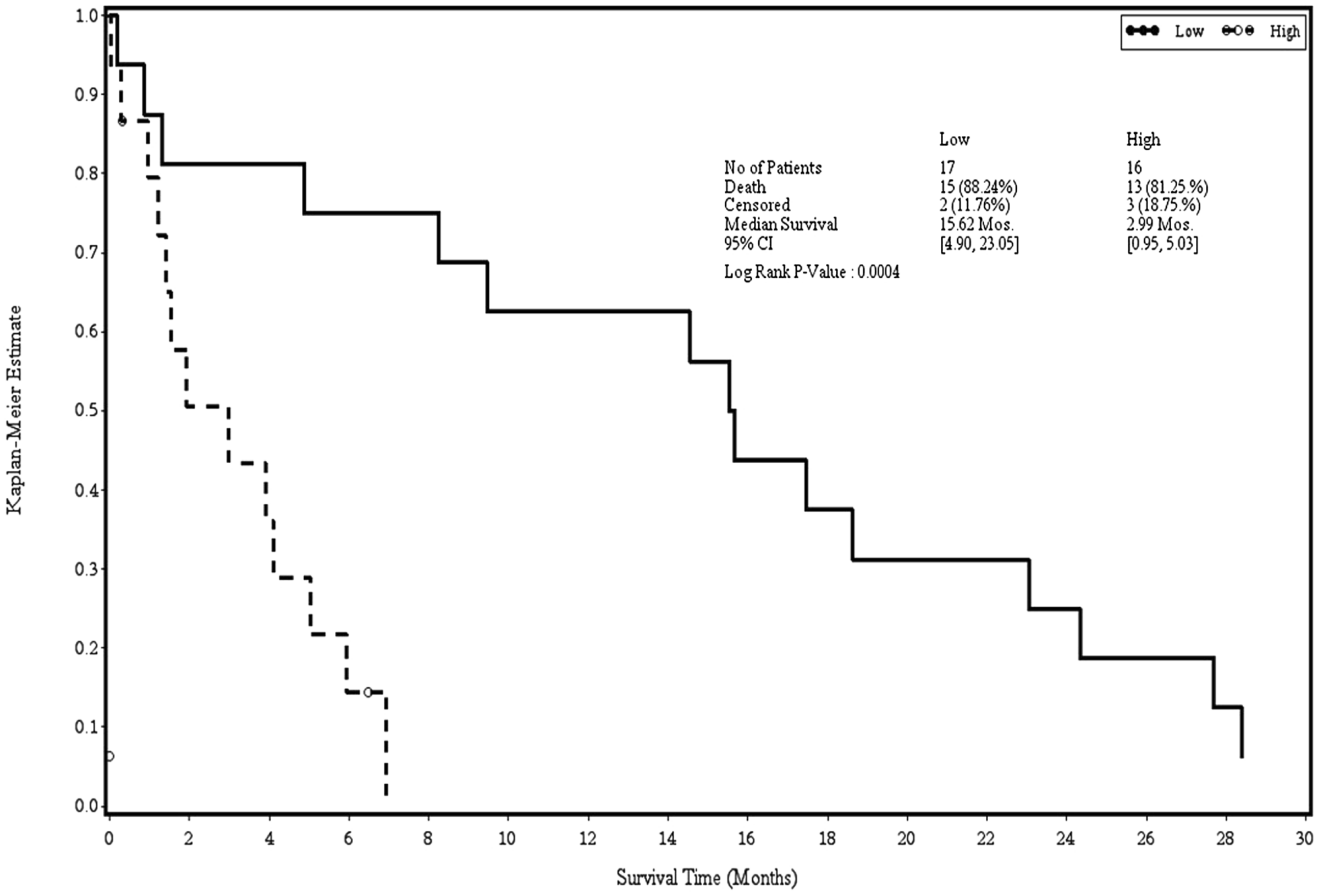
Kaplan–Meier curve for the low-risk and high-risk groups derived from the multivariate Cox model.

Table 5 lists the hazard ratio (HR) from the univariate analysis Cox regression of the association between each expressing gene and survival controlling for sex and age. A gene with HR less than one was regarded as a protective gene, while that with HR greater than one was treated as a risk gene. Among eight genes, only WEE1 was weakly significantly associated with survival. For every unit increase in WEE1, the hazard ratio decreased by 33%. The multivariate Cox regression was assessed to evaluate the association between eight genes and survival controlling for sex and age in Table 6. The backward selection with a stay criterion of 0.2 was used to identify the influential genes. Three genes, WEE1, POLDIP2, and NF1, were selected. WEE1 was the most important protective gene among the three selected genes, followed by NF1. For every unit increase in WEE1, the hazard ratio decreased by 41%. NF1 was weakly associated with survival. Based on the estimates derived from the multivariate Cox regression, a risk score for each patient was computed for the gene expression weighted by the estimates. Patients with a risk score greater than the median were classified into a high-risk group, while those with a score less than the median were classified into a low-risk group. Figure 3 displays the KM curve for two groups. The overall survival was significantly different between the two groups. The median survival time for the high-risk group was only 2.99 months, while that for the low-risk group was 15.62 months.

**Table 5:** Bivariate analysis based on the Cox model.

Variable	Hazard ratio (95% CI)	P
EIF2S3	1.00 (0.56–1.78)	0.996
MDM2	0.72 (0.45–1.16)	0.178
GRB2	1.21 (0.80–1.84)	0.359
WEE1	0.67 (0.44–1.00)	0.051
POLDIP2	0.95 (0.43–2.07)	0.897
RNF4	0.94 (0.47–1.89)	0.867
NF1	0.62 (0.34–1.15)	0.131
DUSP6	0.96 (0.67–1.36)	0.805

^$^Controlling for sex and age.

**Table 6:** Multivariate analysis based on the Cox model.

Variable	Hazard ratio (95% CI)	P
WEE1	0.59 (0.37–0.93)	0.022
POLDIP2	2.24 (0.84–6.02)	0.109
NF1	0.46 (0.21–1.02)	0.056

^$^ Controlling for sex and age.

## Discussion

In this study, we investigated the expression levels of eight NSCLC-associated genes in the pleural effusion of patients with malignant and benign pleural effusion. Out of the eight cancer-related genes, we identified that pleural fluid GRB2, WEE1, RNF4, and DUSP6 mRNA levels were significantly different between patients with MPE and benign PE by using two-independent sample t-tests. In addition, the logistic analysis identified WEE1, MDM2, RNF4, and DUSP6 as MPE-associated factors with high significance. Based on the odds ratio (OR) of these four genes, an effusion with a high expression level of WEE1 and MDM2 and a low expression level of RNF4 and DUSP6 is most likely an MPE case. Moreover, we identified three survival-associated genes (WEE1, POLDIP2, and NF1) associated with overall survival time.

Pleural effusion cytology for MPE diagnosis is only about a 60% positive rate. Therefore, many additional tools have been developed to improve diagnostic power and avoid invasive methods. Traditionally, pleural fluid pH, lactate dehydrogenase, and adenosine deaminase are used for MPE diagnosis^23^ Conventional cancer biomarkers such as CEA, CA125, NSE, CA15-3, and CA19-9 have been intensively investigated to increase MPE diagnostic accuracy^9^. Also, RT-PCR techniques or the detection of cancer cells in pleural effusions have been demonstrated to be more sensitive than immunocytochemistry^14,24^.

Our four-gene logistic model under controlling age and sex provided a sensitivity of 76.8% and a specificity of 89.8% to detect MPE. Nearly 50% of MPE cases had a very high-risk score (greater than 0.9), approaching the maximum of 1.0, while 59.2% of controls had a risk score less than 0.2, very close to the minimum zero. These results indicate that the MPE and benign have distinct gene signatures. In clinical practice, most MPEs were obtained from patients with advanced-stage diseases who received sustained treatments. Either radioactive or chemotherapy of MPE cases might result in the dynamic change of the gene expression in effusion cells and thus the moderate sensitivity for the four-gene model. Nevertheless, our MPE subgroup study showed the advantage of the four-gene model with high sensitivity (100%) to detect pathologically negative effusions whose cells presented as its primary cancer-specific gene expression profile. On the contrary, the antigens in the effusion cells for IHC might probably be less influenced by the chemotherapy on patients.

The four genes included in our model are mainly involved in regulating the cell cycle process. WEE1 is a G2 checkpoint protein kinase that negatively regulates the G2/M checkpoint and plays a role in DNA damage repair and replication stress response^25^. MDM2 proto-oncogene (MDM2) gene encodes a nuclear-localized E3 ubiquitin ligase which could enhance tumor formation by influencing tumor suppressors, such as p53^26^. Ring finger protein 4 (RNF4), a SUMO-targeted E3 ligase, could promote DNA double-strand break repair^27^ Furthermore, RNF4 and MDM2 are involved in DNA damage response and genome stability^28,29^ Moreover, dual-specificity phosphatase 6 (DUSP6) is a member of the mitogen-activated protein kinase (MAPK) family and is involved in controlling the initiation of the cell cycle, especially the G1/S transition^13,30,31^ Based on the known biological functions of these genes, we propose that cells between MPE and benign pleural effusion have significant differences in the regulation of proliferation activities which can be used for MPE diagnosis.

In terms of the overall survival rate of NSCLC, a recent report indicates that an increased number of involved metastatic organ systems and liver metastases are associated with poor prognosis and reduced survival rate^4^. Specifically, they further suggest that advanced NSCLC has a worse survival chance once liver metastases or greater than four metastatic sites are present^4^. In patients with metastatic NSCLC under PD-1 inhibitors treatment, serum lactate dehydrogenase and C-reactive protein concentrations were significantly associated with overall survival^32^. In a recent study, the expression of labyrinthin, a novel cancer neoantigen, was suggested to be associated with prognosis in NSCLC patients^33^. Moreover, the elevated transcription of CPEB4, or IRF4, in peripheral blood was associated with poor survival^34^. We discovered three survival-associated genes from MPE in this study.

The small sample size is the most significant limitation of this study. Selection bias contributes to the low varieties of MPE because about 85% of MPE cases were patients with NSCLC and very few with other cancer types. The composition of the MPE sample is quite different from those in everyday practice. For the survival analysis, due to the lack of treatment information, the Cox regression analyses were only controlled for sex and age. In addition, more MPE cases without any treatment regimen are needed for the study to confirm the diagnostic utility for effusions with cytology negative.

In conclusion, we identified four significant MPE-associated genes in the study, including DUSP6, MDM2, RNF4, and WEE1. The four-gene model had an excellent performance in classifying MPE and benign pleural effusion. In addition, it has 100% sensitivity to detect effusion without pathological confirmation. Thus, this gene panel is a potential candidate for screening MPE in patients with pleural effusion. Furthermore, we also identified three potential survival-associated genes: WEE1, POLDIP2, and NF1.

## Methods

### Patients and Samples of pleural effusion

Patients for the study of molecular markers in pleural effusion (PE) were enrolled in a prospective investigational protocol between July 2017 and December 2018. This study was approved by the Tri-Service General Hospital Institutional Review Board (IRB NO: TSGH-2-105-05-131). All methods were carried out in accordance with relevant guidelines and regulations. The PE samples were collected after obtaining written informed consent from patients at the Tri-Service General Hospital, National Defense Medical Center, Taipei, Taiwan, ROC. Malignant pleural effusions were defined by the presence of malignant cells on cytological examination. This study classified patients with negative cytologic tests, but pleural nodules on computed tomography were also classified as MPE. Non-malignant pleural effusions included transudates caused by congestive heart failure, cirrhosis of the liver, or nephrotic syndrome.

All PE samples were sent to the laboratory department immediately. Thirty-three MPE samples were obtained from patients with cancer. Among them, twenty-eight samples were cytology positive. Two samples were cytology negative but pleural biopsy positive. Three samples were from patients clinically diagnosed with imaging findings. Effusions, characterized as transudate, served as controls, collected from forty-nine patients with non-malignant diseases caused by cardiac failure, nephrotic syndrome, cirrhosis of the liver, or pulmonary inflammation.

### Preparation of cells from pleural effusion, RNA extraction, and cDNA synthesis

Samples of 50–100 mL pleural effusion (PE) were thoroughly mixed with EDTA to a final concentration of 1.5–2.0 mg/mL and temporarily stored at 4 °C before the cell separation procedure within 3 h of collection. Each fluid sample was centrifuged in a 50 mL tube at 380 × g for 10 min at 4 °C. The cell pellet was then carefully suspended and washed twice using 20 mL PBS containing 1 mM EDTA. The cell pellet was obtained after centrifugation under the conditions above and dissolved in TriPure Isolation Reagent (Roche, Germany) for RNA preparation according to the modified manuscript. Chloroform was replaced with 1-bromo-3-chloropropane (Sigma, Taiwan). The procedures of cDNA synthesis and quality assessment of nucleic acids were according to Chian et al.^20^ and Huang et al.^35^. All RNA and cDNA were stored at − 80 °C before the next analytical steps.

### Quantitative real-time PCR

An SYBR Green-based detection method was applied using pre-designed, gene-specific primer sets purchased from Advpharma (Taiwan) and GoTaq qPCR Master Mix (Promega, USA) on a Roche Cobas z480 (Roche, Germany). The specificity of primers for each testing gene was evaluated on a small number (approximately 10) of PE samples using real-time PCR. These primer sets were experimentally validated with the following criteria: (i) a single melting temperature; (ii) a single gene-specific amplified product was confirmed by DNA gel electrophoresis; (iii) the amplification efficiency ranged between 90 and 95%, and (iv) the Cp-value was less than 30. After this preliminary test, the real-time PCR measurements of the investigating genes fulfilled the abovementioned criteria and were thus applied for further investigation. In this study, expression levels of eight genes (EIF2S3, MDM2, GRB2, WEE1, NF1, POLDIP2, RNF4, and DUSP6) and the reference gene HPRT1 (Hypoxanthine Phosphoribosyltransferase 1) were analyzed. According to our previous reports, these eight genes were highly correlated with NSCLC^20^. The expression of each investigated gene in a sample was normalized to that of the HPRT1 gene and presented as a delta Cp value (Cp[HPRT1] − Cp[studied gene]), which is inversely correlated with the gene expression^36^.

### Statistical analysis

The gene expression levels were evaluated in PE samples and were summarized by the mean and standard deviation (Stddev). The Chi-square and two-independent sample t-tests were first used to assess bivariate associations between demographics and gene expressions in two types of effusions. Second, the logistic regression was conducted to determine the association between gene expression and the status of malignant cells controlling for demographics. In particular, the backward selection with criterion 0.2 was used to remove insignificant predictors. The model of fit was assessed by the ROC curve (receiver operating characteristic curve) and the C statistic (area under the ROC curve; AUC), and the level of impact of the covariates was evaluated by the standardized coefficients (StdEst). Models are typically considered reasonable when the C statistic exceeds 0.7 and strong when C exceeds 0.8^37^. The ROC curve determined the cutoff value for classifying a sample with or without malignant cells according to sensitivity and specificity. Furthermore, the overall accuracy, positive predicted value (PPV), and negative predicted value (NPV) were also obtained for the given cutoff value. In addition, the risk scores of malignant cells were computed for the best model and ranged from zero to one. Finally, a histogram of the risk scores according to MPE cases and controls was plotted with an interval of 0.1.

The univariate association between each gene expression and overall survival controlling for sex and age was assessed by the Cox model. The same model also evaluated the multivariate association between 8 gene expressions and survival. To avoid overfitting, the backward selection with an exclusion criterion setting to 0.2 was used to select the most influential gene expressions. The sum of estimated coefficients multiplied by the corresponding gene expressions was computed to represent a patient’s risk score. Patients were classified into the low-risk or high-risk groups according to the risk score, with the sample median of the risk score as the cutoff value. The model’s predictive power was assessed by computing the HR between the high-risk and low-risk groups, which was obtained again by the univariate Cox model.

## Data Availability

The datasets used and/or analyzed during the current study are available from the corresponding author on reasonable request.
